# Optimization of Work Environment and Community Labor Health Based on Digital Model—Empirical Evidence from Developing Countries

**DOI:** 10.3390/ijerph192013114

**Published:** 2022-10-12

**Authors:** Shiya Gao, Zeyu Wang, Shaoxiang Jiang, Wen Ding, Yuchen Wang, Xiufang Dong

**Affiliations:** 1 School of Management, Wuhan Polytechnic University, Wuhan 430023, China; 2 School of Public Administration, Guangzhou University, Guangzhou 510006, China; 3 National School of Development, Peking University, Beijing 100871, China; 4 Faculty of Engineering, University of Waterloo, Waterloo, ON N2L 6J5, Canada; 5 Carey School of Business, Johns Hopkins University, Baltimore, MD 21202, USA

**Keywords:** FCEM manufacturing enterprises, enterprise digital transformation, health and safety management, internet of things, big data technology, FCEM

## Abstract

As far as we know, for large manufacturing enterprises, there is often a community of labor gathered around such enterprises, which is especially used as a place for the enterprise to place the labor force. This paper aimed to update the industry model of Chinese Manufacturing Enterprises (CMEs) to improve workers’ health management. This work first discusses the value, mode, and process of Enterprise Digital Transformation (EDT) and Worker Health and Safety Management (WHSM). Then, it proposes the CMEs-oriented EDT model and WHSM system based on Big Data Technology (BDT) and the Internet of Things (IoT). The proposed model and system are verified through a case study on the Shanghai BYD manufacturing enterprise (short for BYD) using the Fuzzy Comprehensive Evaluation Method (CFEM). The EDT model verification considers the adaptation and performance of enterprises after EDT. The WHSM system considers workers’ oxygen inhalation status to evaluate their heart and cardiovascular health. The results show that EDT improves the enterprise’s revenue and reshuffles the revenue structure. The EDT model has absolute adaptability to BYD. It has greatly improved BYD’s indexes, especially financial performance, market capability, and technical capability.

## 1. Introduction

Chinese Manufacturing Enterprises’ (CMEs) profit margin is still meager compared with developed nations. Enterprise Digital Transformation (EDT) faces many difficulties, such as a lack of capital investment [1]. First, the CME executives are not aware of the necessity, urgency, and complexity of EDT, and the management concept stays on the deployment of common Information Technology (IT) systems. The IT department cannot achieve Enterprise Digital Transformation (EDT) alone. It must be led by the enterprise decision-makers and promoted from top to bottom [2,3]. Secondly, CMEs have applied many information systems. However, the basic data are inaccurate, and the coding system is not unified, failing to promote the EDT [4]. Many CMEs believe that promoting an automation system can accelerate the effect of EDT, and there is a general situation of emphasizing automation and neglecting digitalization. Additionally, many CMEs only emphasize production line automation and labor reduction without a solid foundation for equipment networking and data acquisition. The workshop has not genuinely realized visualization, failing to monitor equipment and manage workers’ health conditions. Moreover, the CMEs-oriented EDT investment has not achieved remarkable results, restricting their motivation to further transformation. For example, some CMEs invest in self-built e-commerce platforms. Nevertheless, most customers still favor mainstream e-commerce platforms, so self-built platforms have not achieved the expected results. Meanwhile, sub-industries under manufacturing vary greatly. Enterprises from different industrial chains feature high personalization. Thus, no universal implementation path is applicable, and no ready-made EDT models are available [5,6].

In March 2022, Zhengzhou Haier Water Heater Interconnection Factory (Zhengzhou Haier) was selected by virtue of the wide application of advanced industrial 4.0 technology in the whole industrial chain, becoming the world’s first end-to-end “lighthouse factory” of water heaters and the fourth landing application of the Haier Group. The World Economic Forum (WEF) commented: “facing the vigorous development of the water heater market and the increasing demand for high-end products and services, Zhengzhou Haier, a new factory, uses Big Data Technology (BDT), and 5th Generation (5G), Edge Computing (EC), and ultra-wideband solutions to establish close ties with suppliers, factories, and users.” From 2020 to early 2021, Haier’s water heater’s order response speed increased by 25%, production efficiency increased by 31%, and product quality increased by 26%. According to the report of the WEF, each lighthouse factory has unique and diversified success cases. More broadly, it mainly includes the digital realization within a single factory (focusing on digital assembly, processing, maintenance, performance management, quality management, and sustainable development) and opening up the end-to-end value chain (focusing on the supply network, product development, planning, delivery, and customer connectivity). Obviously, EDT has brought a new business model to Zhengzhou Haier, improved its core competitiveness, reduced operating costs by digital means, increased revenue and efficiency, and upgraded management mode and operation efficiency [7].

In fact, EDT uses digital technology to promote a series of changes in production mode, organizational structure, and corporate culture [8]. After EDT, enterprise management should explore new sources of revenue and develop new products, services, and business models. Therefore, EDT is the deep integration of technology and business models, and it promotes digital technological development and technical support capabilities. EDT revolutionizes the traditional business model by creating a new and dynamic digital business model [9,10]. In terms of the research on EDT, Zimmerman et al. (2019) studied the transformation and upgrading of enterprises based on GVC. They proved that cross-industrial upgrades could improve enterprises in all aspects [11]. Kaletnik et al. (2020) explored the method and process of EDT and put forward a new path and implementation EDT scheme [12]. Correani et al. (2020) examined EDT and upgrading from internal and external aspects [13]. Shen et al. (2021) believed that innovative technologies could be used to realize the CMEs-oriented EDT [14]. Meanwhile, Kane et al. (2019) argued that the EDT could realize the data connection between all manufacturing enterprises and the digital structure up-gradation and improve operation efficiency [15]. Son et al. (2019) contended that changing enterprise operation thinking could promote EDT. For example, the new operation modes could be realized by expanding consumer groups [16]. The above literature carries out EDT aiming at a certain point of the enterprise business model. They do not comprehensively study the enterprise organizational structure, enterprise resource allocation method, production, and operation mode. Thus, the above research is not comprehensive and mono-directional.

At the same time, in recent years, workers’ sub-health and even sudden death have attracted much attention. All social sectors generally call for strengthening Worker Health and Safety Management (WHSM). Workers have promoted the rapid development of enterprises. However, the problem of overwork also affects workers’ physical and mental health. The claim settlement data of corporate group insurance also presents the younger trend of workers’ illnesses. The 2021 Group Health Insurance Claim Settlement Report released by Taikang Endowment Insurance Co., Ltd. (Beijing, China) shows that 54% of workers with hypertension are young adults aged 30–49; 45% of workers with intervertebral disease are concentrated in middle-aged adults, 30–39 years old; and 43% of sudden death cases are concentrated among young people in the 40–49 age group. In fact, most sub-healthy people often do not realize what risks they are facing. They often need to go to the hospital to realize the seriousness of their health problems. This traditional concept of emphasizing “medicine” over “prevention” easily leads to workers’ health status deterioration, increases the medical expenses of enterprises and society, and exacerbates the shortage of medical resources. Therefore, enterprise managers gradually have formulated strategies for WHSM.

The keywords of health management involve many aspects of research. At present, the actual development of health management, especially in community health services, WHSM, and the health insurance industry, still lacks systematic research and governance [17]. Therefore, this work applies the Internet of Things (IoT) technology to establish the CMEs-oriented EDT model and WHSM system. The aim is to provide solutions for the CMEs’ development and promote workers’ health conditions.

Firstly, this work discusses the value, mode, and process of EDT and WHSM in CMEs. Secondly, based on the BDT and IoT, it establishes the CMEs-oriented EDT model and the WHSM system. Finally, Shanghai BYD Manufacturing Enterprise (short for BYD), it evaluates the adaptation and performance of the proposed EDT model through Fuzzy Comprehensive Evaluation Method (FCEM). Under the proposed WHSM system, workers’ health is analyzed by considering their heart and cardiovascular situation when inhaling hyperbaric oxygen.

## 2. Materials and Methods

### 2.1. Significance of EDT

**Figure 1:**
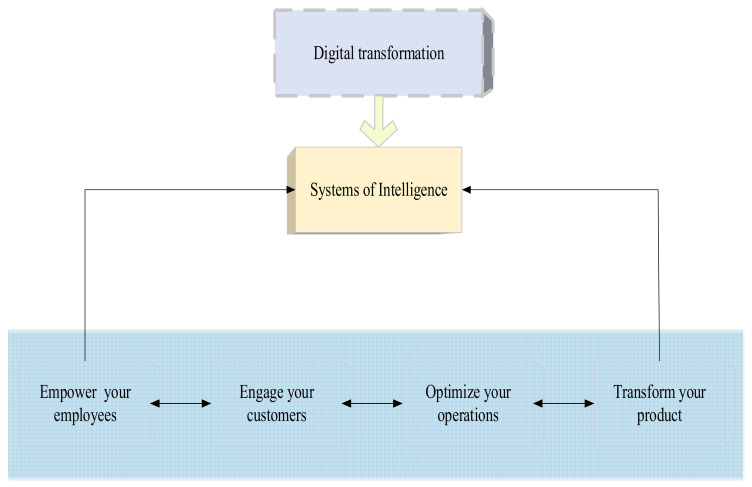
Value system of EDT.

Both “digitization” and “digitalization” are used in EDT. The first term means explicitly converting analog information into digital information (for example, manually filled documents are automatically recognized as digital information). The other term refers to integrating digital information into enterprises, deepening the application of various business software and emerging technologies, such as the IoT. In the research process, it is believed that the digital transformation of manufacturing enterprises is not a single-link improvement process, but more like the penetration of digital kinetic energy into all components of manufacturing enterprises to stimulate the aggregation effect of enterprises. In this process, it is not to apply the emerging digital technology surface to the production and circulation of products. It not only improves production efficiency, but also reconstructs business processes through new digital thinking concepts, generates new economic benefits, better opens up new markets, and improves the competitiveness of enterprises. The essence is to apply digitalization to the strategic layer of the enterprise, to promote the reform of the organizational structure and management methods of the enterprise, and to enhance the core competitiveness of the enterprise through endogenous and external growth. Feng et al. (2022) defined digitization as the transformation of organizations that integrate mathematical techniques and business processes into the digital economy [18]. Shpak et al. (2020) defined digital transformation as the use of technology to radically improve business performance or reach [19]. It helps realize data-driven decision analysis and completely changes the business process of enterprises. Essentially, EDT is the process of enterprises’ real digitalization. Lin et al. (2020) studied the digital transformation of manufacturing enterprises, and believed that the digital transformation of manufacturing enterprises is not the further improvement of the evolution of various business processes, but a comprehensive reform of the value proposition, operation mode, enterprise organization mode, resource allocation mode, R&D mode, production mode, and marketing mode [20]. Figure 1 is the value system of EDT.

**Figure 2:**
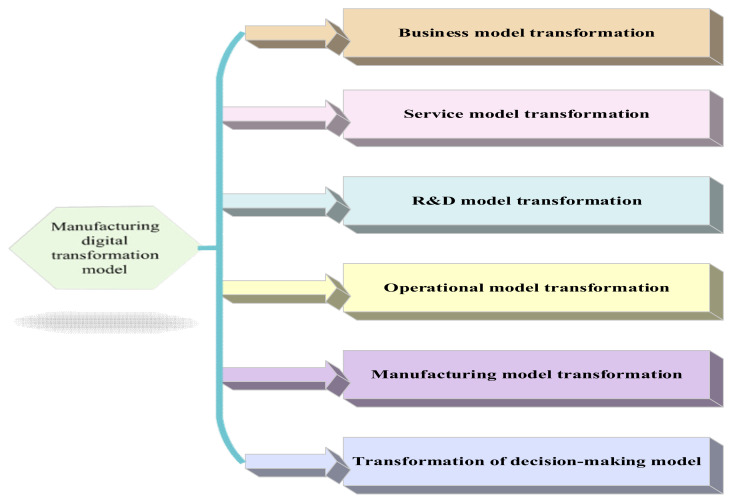
Common modes in EDT for manufacturing enterprises.

In Figure 1, EDT can realize data acquisition, state perception, remote control, increased product added value, and service revenue. At the same time, EDT can help manufacturing enterprises cope with increasingly complex compliance requirements, especially in industries involving people’s livelihood, such as medicine, food, and import–export. EDT helps them realize the traceability of the whole production process. The complexity of manufacturing enterprises’ organization, business, products, and value chain has brought many obstacles to the EDT [21]. Maciag (2022) studied the basic model of digital transformation and believed that digital transformation of enterprises should use the Internet’s way of thinking to reconstruct enterprises from four aspects: business model, capital model, management model, and mental model, so that enterprises can obtain stronger vitality through transformation [22]. Zhang et al. (2022) studied the basic model of digital transformation of Chinese manufacturing enterprises and believed that China’s manufacturing industry had many problems such as excess capacity, low resource utilization, and unreasonable structure. The technology and thinking of the Internet should be used in the whole life cycle of the manufacturing industry. Traditional industries should be transformed into science and technology, intelligence and efficiency, redefine R&D design, manufacturing, operation management, sales and services, and promote the in-depth development of the industry and industrial transformation and upgrading [23]. Figure 2 gives common modes of EDT for manufacturing enterprises. If EDT (Enterprise Digital Transformation) is regarded as a theoretical framework model, we need to define its components. Then, considering the basic theory of digital transformation, it should include changes in the business operation mode, service mode, research and development mode, organizational structure management mode, and production mode. After completing the above changes, the decision-making model of the enterprise will be comprehensively changed.

In Figure 2, the common modes of manufacturing enterprises’ EDT are dissected in detail. First, the new business model is a Pay-For-Service mode based on digital technology. Enterprises no longer sell products but sell the services they use. To realize the Pay-For-Service mode, enterprises must realize product digitalization. The products themselves should be integrated into a Cost Per Sales (CPS) system with communication, calculation, and control capabilities. At the same time, a cloud platform must be established to monitor product operation to achieve predictive maintenance of the operating products. Additionally, manufacturing enterprises should also promote the customization of online products and the combination of online and offline experience marketing, which is also part of the transformation of the business model. Second, the service mode transformation enables customers to realize self-service by developing products and service-specific Applications (APPs) to improve service efficiency. Some of the world’s leading equipment manufacturing enterprises can also develop service APPs to realize remote condition monitoring and predictive maintenance. Using Augmented Reality (AR) technology to maintain the equipment and display the equipment sensor data and the assembly process can greatly improve the efficiency of equipment maintenance. Third, the Research and Development (R&D) model transformation can reduce the testing of actual manufactured products through simulation-driven design. By managing the R&D data and process of the whole product life cycle, enterprises can improve the reuse rate of parts and the R&D efficiency while reducing costs. In addition, it can realize remote and collaborative R&D. Fourth, operation mode transformation can help manufacturing enterprises realize fine management. Fifth, the manufacturing mode transformation can be associated with the technology of other excellent enterprises to realize fully automatic processing of different mechanical parts. Lastly, enterprises can conduct multi-dimensional big data analysis by transforming the decision-making model. It improves the real-time visualization of data analysis. As such, enterprises realize data governance, decision-making based on the data drive, and analyzing the key information behind the data using AI and BDT [24].

**Figure 3:**
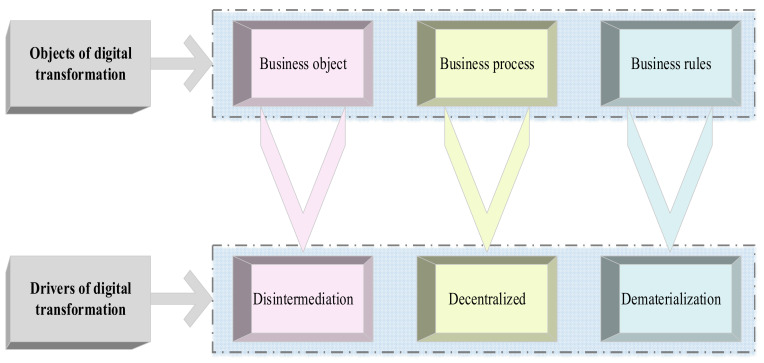
EDT flow of manufacturing enterprises.

In order to promote EDT, manufacturing enterprises must define the digital transformation strategy, formulate the digital transformation plan, and then implement it. In this process, they need to rely on professional consulting service institutions to complete processes such as EDT state diagnosis, demand analysis, process review, and overall framework [25]. Bican et al. (2020) studied the process of digital transformation and believed that digital transformation is a process of upgrading strategic thinking. Although this process may involve the upgrading of enterprise Information Technology (IT) infrastructure, it is not a technology in nature, but a strategy [26]. Figure 3 illustrates the flow of EDT of manufacturing enterprises. After setting the basic elements to be considered for the transformation of the Enterprise Digital Model. In fact, the figure defines the object of digital transformation from the perspective of goal setting, and analyzes the motivation and necessity of digital transformation from the perspective of Motivation Analysis Theory.

### 2.2. CME and WHSM

**Figure 4:**
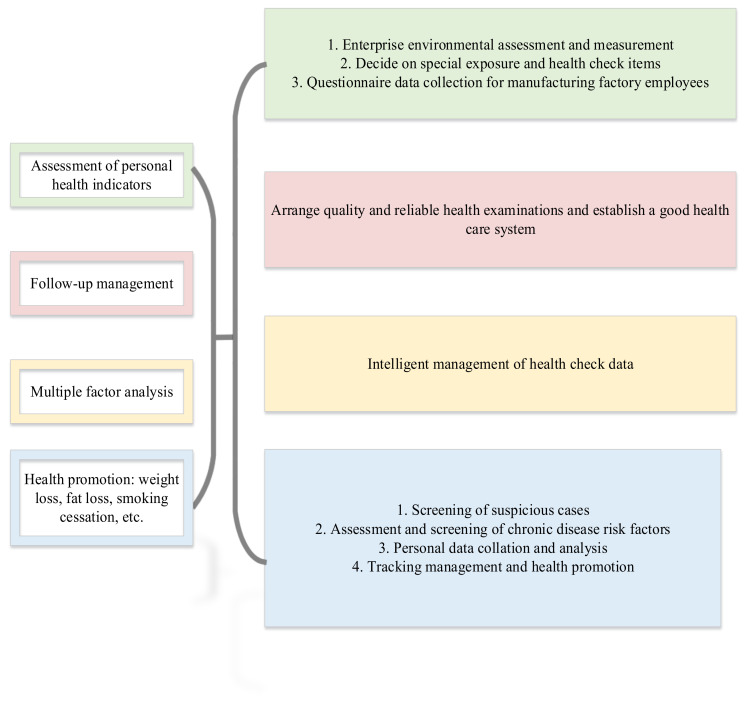
Process of WHSM in manufacturing enterprises.

According to the 2020 China Workplace Worker Health Risk Report survey results, 29% of workers are overweight and obese, 43% have substandard diets, and 48% lack sleep. Among these respondents, more than 60% believe that their sub-health status affects their normal life and activities, and more than half of the workplace workers lack vitality [27]. It shows that the enterprise’s good employee health management is a necessary guarantee for the development of the enterprise. Whether it is to ensure the growth and development of enterprises and build harmonious labor relations from the macro level of national policies, social economy, and meso level, or from the perspective of the health needs of employees and their families at the micro level, it is the general trend for enterprises to do a good job in employee health management. The research believes that the concept of personal mobile health management is a way to monitor and record the whole process of human health by using mobile network technology. Mobile health includes smart terminals, health data management, related matching resources, and collaborative systems. Whether it is a pure digital medical concept or a health management IoT architecture based on the IoT and cloud computing, its core business is still in the field of mobile health management. There are four main points in mobile health: First, is the collection of human data. Some supplementary programs are related to human health and fitness, and others do medical consultations that do not require a lot of diagnosis and treatment methods and data. Follow-up can be performed after discharge, including disease investigation and remote testing. Priyono et al. (2020) integrated the resources and transformation strategies of enterprise digital transformation, and proposed a theoretical framework of resource adaptation for enterprise digital transformation. They believed that the prerequisite variables for the success of the digital transformation of enterprises are internal and external resources and internal and external capabilities, and external capabilities are an important factor for the success of digital transformation [28]. The attention to enterprise workers’ physical and mental health has also been improved. Managing workers’ health and welfare has become an important consideration for many people’s career choices. Figure 4 describes the process of WHSM in manufacturing enterprises. Figure 4 is in Figure 3. On the basis of the Business Process mentioned above, specific examples were analyzed. Further, we analyzed how Business Process is designed and works in WHSM based on our research objectives.

### 2.3. IoT Technology

**Figure 5:**
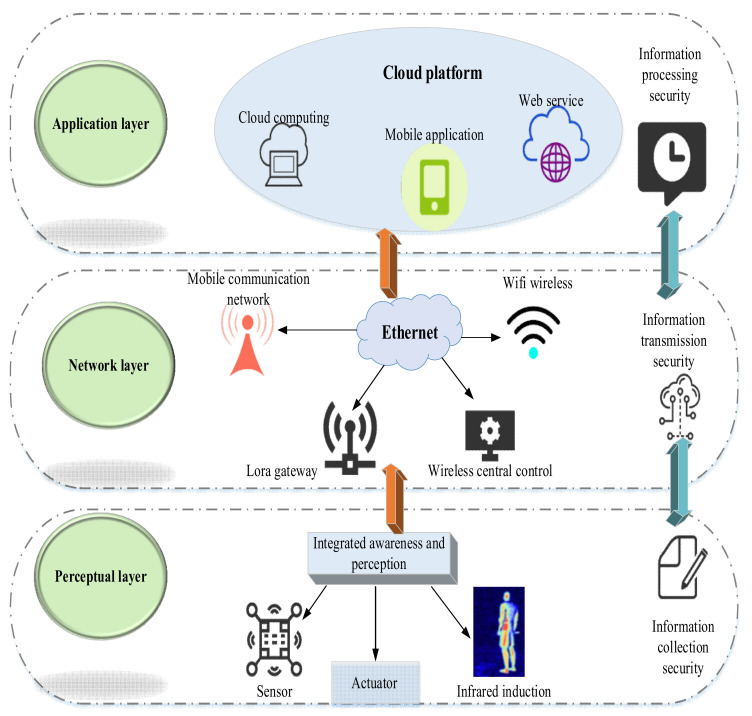
IoT+cloud platform architecture.

With the development of Cloud Computing (CC), BDT, and other IT, the IoT+cloud platform has come into being. It is the value cohesion of the IoT industry. Moreover, the IoT+cloud platform sees a vast market potential with more technological innovation. The IoT+cloud platform is currently in the exploration of post-precipitation mode. It is about to enter the inflection point before rapid development, from focusing on the underlying hardware to the multi-scenario business capability of the software platform [29]. Mehmood et al. (2019) studied the characteristics of the IoT platform and believed that the core and foundation of the IoT was still the Internet, which was an extended and expanded network based on the Internet. The IoT client extends to any item for information exchange and communication [30]. Figure 5 demonstrates the architecture of the IoT+cloud platform. Figure 5 is the link to the product design. For example, based on how IoT plays a specific role in digital transformation, especially in the digital transformation of manufacturing enterprises. This step is equivalent to the design of the transformation path based on the theoretical design in Figure 2, Figure 3 and Figure 4 and the actual situation.

According to Figure 5, the IoT+cloud platform connects and manages IoT terminals through the network and perception layers. It collects and stores sensing data and provide standard interfaces and general tool modules for development and applications through the network and application layers. Finally, it indirectly reaches the end-user through Software as a Service (SaaS). The IoT+cloud platform is the technical integration of the IoT platform and CC. From the perspective of the concept of the IoT, the concept of the IoT must be the process control of the whole process, which must be supported by standards. Evolution is the use of complex IT technology to create a simple digital health management system. The standardization of the digital health management system is to improve the standardization level of the digital health management system and gradually promote the standardization of the digital health management process. Standardization of digital health management systems also solves IT challenges. The most difficult thing for manufacturing companies is the health management of employees. There is no perfect standard for employee health management. The emergence of the IoT essentially promotes the improvement of the entire medical informatization, and the digital health management system has broad application prospects in the development of enterprises.

Cloud computing is also a way to achieve infrastructure sharing, where a large number of resources are linked together in a public or private network to provide IT services. From an IT perspective, cloud computing is the provision of Internet-based software services. The software used by the user does not need to be on their own computer, but uses the Internet to access the software on an external machine through a browser. All work, user files, and data are also stored on these external machines [31]. Big data, on the other hand, enable the collection of raw, structured, or unstructured data from various sources. With the execution of machine learning techniques and big data predictive models, it is feasible to predict machine failures and customer behavior. Big data analytics helps businesses identify customers by understanding their thought processes and feedback in advance. Enterprises can adjust their strategies accordingly, providing deeper insights from disparate data sources in unique ways to help define the overall strategy. Big data analysis can promote customer acquisition and retention, effectively manage enterprises, and reduce enterprise management risks [32].

The large-scale application of edge computing can improve computing efficiency, reduce latency, and improve data security. In this way, various problems caused by the excessively centralized data processing mode of cloud computing in the current digital transformation can be solved. Massive amounts of data for digital transformation are aggregated from tiered networks to the cloud, where they are then analyzed and processed. In the era of massive data generation, the efficiency of this data processing method will become lower and lower. Sinking data to the “micro cloud” on the edge side for processing can not only improve efficiency, but also promote the transformation of traditional manufacturing from “hardware thinking” to “service thinking”. However, edge computing drives digital transformation from product-oriented to service-oriented. Every terminal with edge computing capabilities can become a service provider, and all service providers are on the same plane. The application of edge computing in digital transformation drives the transformation of the life cycle from product to service operations, which in turn leads to product service and business model innovation. This has a profound impact on the development of value chains, supply chains, and ecosystems, and promotes their high-quality development [33].

#### 2.3.1. Modeling CMEs-Oriented EDT Based on IoT

Applying IoT in manufacturing enterprises is called Manufacturing Internet of things (MIoT). It focuses on Machine to Machine (M2M) communication. It uses BDT, AI, CC, and other technologies to achieve more efficient and reliable manufacturing operations.

As an information technology term, big data can refer to the data set. These data sets are difficult to process and analyze by traditional computing methods. However, they can be systematically obtained by advanced technical means, including collection, storage, analysis, transmission, retrieval, and display. They can generate value. Therefore, big data is a collective term involving both the data sets and corresponding processing methods. The distributed file system is the first link in the big data storage system. Its basic idea is to establish a file system on multiple machines using the master–slave structure. The file system exposes the management functions and interface capabilities through the master node. The real data are stored on the data node. However, all read and write operations are scheduled through the master node. The file system has multiple backups to avoid data loss or single-node failure errors. Multiple backups can also be used for load balancing between nodes. Another type of big data storage is relational database clusters. The traditional relational database system includes two most important parts, the storage engine, and the Structured Query Language (SQL) service. The storage engine is responsible for data storage and reading. SQL services include SQL parsing, caching, and index calculation. The stand-alone version of the relational database is limited by the size of the stand-alone storage space. In contrast, the cluster version of the relational database can handle large-scale data sets. The storage engine is the key to breaking through the single-machine storage capacity. It can support multiple data backups and share data among multiple SQL service instances [34].

**Figure 6:**
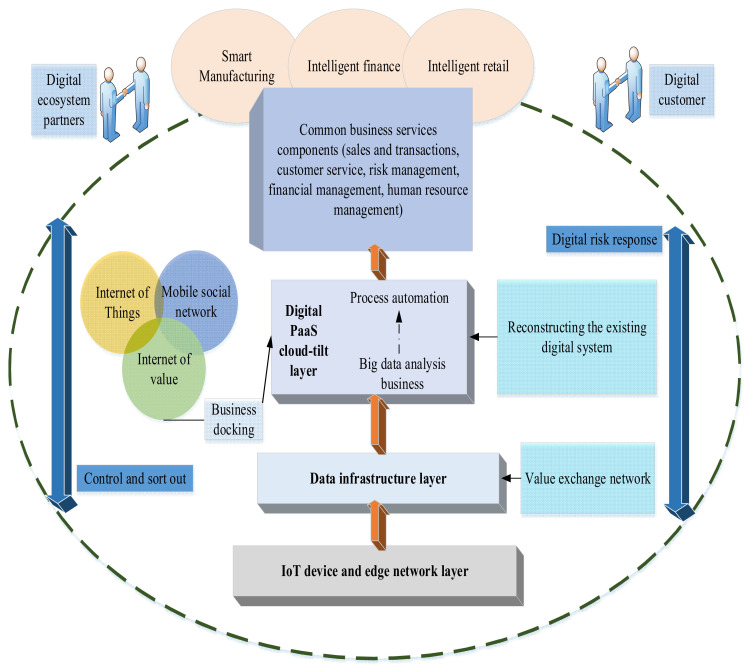
CMEs-oriented EDT model based on IoT.

Big Data Analysis (BDA) plays a key role in the preventive maintenance of production equipment. Its core is the cyberspace entity system. The network entity system is designed through the 5C (Computer, Communication, Content, Customer, and Control) framework to realize the connection, transformation, networking, cognition, and configuration. It converts the collected data into useful information and optimizes the production process. Traditionally, service providers mainly obtain solutions through experiences in EDT, only referring partly to data and process analysis. However, the traditional EDT services are no longer applicable in the digital era, and many experiences and data connotations have changed quietly. Establishing a digital ecosystem is completely different from traditional informatization [35]. Willner et al. (2020) studied the core technologies of enterprise digital transformation, and believed that digital technology could improve the output or production technology of manufacturing enterprises, thereby realizing the improvement of the enterprise economy. The application of digital technology can link the data islands among all levels of the industry, improve the operational efficiency of enterprises, and build a new pattern of digital economy [36]. Figure 6 depicts the proposed CMEs-oriented EDT model based on the IoT.

Figure 6 provides a common CEMs-oriented EDT model built around vision, data, and organization. A comprehensive and systematic organizational capability change and transformation model will be completed by coordinating the strategy formulation, business functions, data, and implementation. Figure 6 is mainly based on Figure 5. The proposed IoT design is combined with the concept of CMES. This is mainly based on the analysis of the theory of information asymmetry between customers and enterprises. The reason why information asymmetry between them is greatly reduced by introducing CMES and IoT technology.

#### 2.3.2. WHSM System Based on IoT

**Figure 7:**
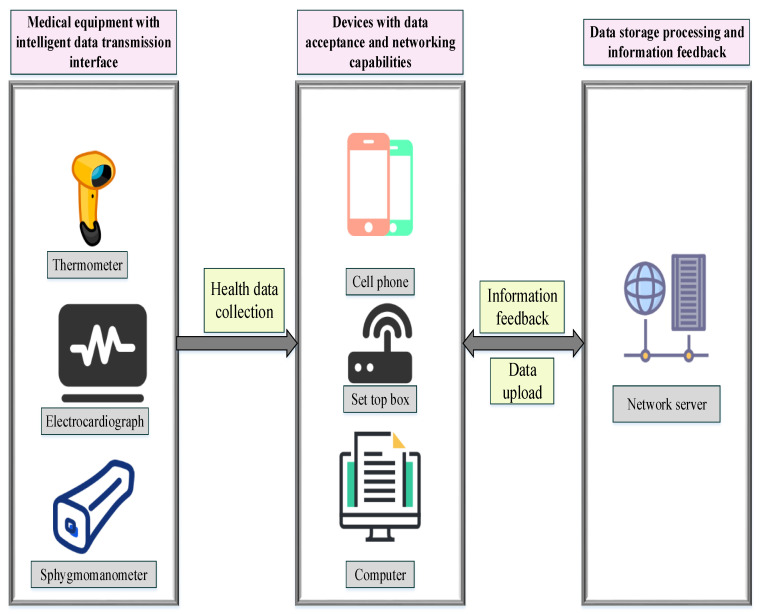
WHSM system based on IoT.

WHSM helps enterprises comprehensively monitor workers’ health risk factors. Manufacturing enterprises can apply IoT-based visualization edge gateway, integration unit, micro base station, IoT Access Point (AP), and modules. In particular, visual edge gateways are used in low-frequency proximity sensing and positioning and long-distance signal transmission coverage in public areas. All units and departments use base stations and IoT modules (that support equipment access to IoT platforms with different frequency bands and protocols). These modules can expand the IoT to meet business needs with various application scenarios. Dayan et al. (2022) studied the development of health management systems and found that most pharmaceutical companies have established a standard system of equipment, frequency, operation, and coding for their own use. The standardization and compatibility are poor, affecting applications outside the system and within the supply chain. The IoT technology industry, which is closely related to health management, is lagging behind in China. Chip design and manufacturing, antenna design and manufacturing, label packaging and packaging equipment manufacturing, reader device development, data management software design, and other production links are a complete production process. In the IoT technology industry chain, there is a shortage of talent in RFID-related technology development, system design, experimental testing, and project implementation, and training is urgently needed [37]. By building a healthy MIoT environment, enterprises can invite more partner enterprises and avoid repeated construction. Moreover, it can simplify the network system architecture and reduce ownership and maintenance costs. The proposed CMEs-oriented WHSM system based on IoT is displayed in Figure 7.

As in Figure 7, the WHSM is an information management system that tracks, records, analyzes, and processes the personal health parameters of enterprise workers over the long term. It feeds back workers’ health suggestions on time. The system is based on the IoT technology and the Internet server and runs for a long time. Meanwhile, the health data of the employees of the enterprise is continuously updated on the Internet terminal, and the ability to analyze and process the data is improved to more comprehensively monitor the personal health of the enterprise labor force, provide more complete health guidance suggestions, and achieve the purpose of preventing working diseases and protecting the health of the labor force. Finally, as seen in Figure 7, the WHSM system based on IoT is actually the final result of the whole model design. Through analysis, we summarized s the types of IoT devices commonly used in Chinese manufacturing enterprises and how to incorporate these devices into a design framework of the WHSM system. This design module analysis diagram is actually a summary of the above logic derivation.

### 2.4. Data Source and Processing Method

Taking Shanghai BYD Manufacturing Company as an example, this paper analyzed the adaptability and effect of the enterprise’s digital transformation, as well as the health management of employees after the enterprise’s digital transformation. Due to the need to measure the effect of digital transformation on manufacturing enterprises from many aspects, it is difficult to determine the measurement standard among various indicators of the enterprise. Therefore, the Fuzzy Comprehensive Evaluation Method (FCEM) is adopted to evaluate the adaptability and effect of digital transformation of enterprises [38,39]. FCEM is a comprehensive evaluation method based on fuzzy mathematics, which comprehensively evaluates things or objects restricted by many factors. It has the characteristics of clear results and strong systematicness. It can better solve problems that are fuzzy and difficult to quantify, and it is suitable for solving various uncertain problems. It is assumed that there are n factors related to the evaluated indexes, and the index set is given below:

(1) $$U=\left\{\left.u_{1},u_{2},\cdots ,u_{i},\cdots ,u_{n}\right\}\right.$$

In (1), U is the total index factor. $u_{i}$ indicates the single-index factor. The weight $A=\left\{a_{1},a_{2},\cdots ,a_{n}\right\}$ is used to measure the importance of each factor. Set m shows the possible comments. (2) describes the comment set:

(2) $$V=\left\{v_{1},v_{2},\cdots ,v_{i},\cdots ,\left.v_{m}\right\}\right.$$

In (2), V is the total comment set. $v_{i}$ represents a single comment on an index. The membership vector $r_{i}=\left(r_{i1},r_{i2},\cdots ,r_{im}\right)$ is obtained by single factor evaluation, finally forming a membership matrix, as shown in (3):

(3) $$R=\left[\begin{array}{cccc} r_{11} & r_{12} & \cdots & r_{1m}\\ r_{21} & r_{22} & \cdots & r_{2m}\\ \vdots & \vdots & \vdots & \vdots \\ r_{n1} & r_{n2} & \cdots & r_{nm} \end{array}\right]$$

Then, we determined the factor centralization vector, normalized the evaluation set, and finally calculated the comprehensive evaluation vector. The purpose is to make the comprehensive evaluation value according to the principle of maximum membership. The calculation of the comprehensive evaluation vector is shown in (4) (where ∘ is a fuzzy operator):

(4) B=A∘R

Based on this, a Comprehensive Fuzzy Evaluation (FCE) was conducted by factoring in the five indexes of the enterprise after the EDT. Specifically, the five indexes are enterprise operation (financial performance), market capacity, technical capacity, management capacity, and enterprise environment. Then, the comment sets $v_{1}$, $v_{2}$, $v_{3}$, and $v_{4}$ represent very adaptable, relatively adaptable, not much adaptable, and totally unadaptable. The membership vector is obtained by evaluating a single index. According to the capital distribution of BYD, the weight of the index vector is set as $\left\{0.3,0.2,0.3,0.2,0.1\right\}$.

In recent years, there have been more and more sudden death and stroke among manufacturing workers. After health management, the health status of workers is measured by the oxygen uptake of workers [40]. The calculation of blood oxygen saturation is shown in (5):

(5) $$SpO_{2}=HbO_{2}/(HbO_{2}+Hb)\times 100\%$$

In (5), $HbO_{2}$ is oxygenated hemoglobin. Hb represents the hemoglobin in red blood cells. The capability of blood to carry and transport oxygen is measured by oxygen saturation. Clinically, the f oxygen absorption concentration is calculated by (6):

(6) Inhaled oxygen concentration(%)=21+4×Oxygen flow(L/min)

## 3. Results

### 3.1. Adaptability and Performance Analysis of CMEs-Oriented EDT Model

**Figure 8:**
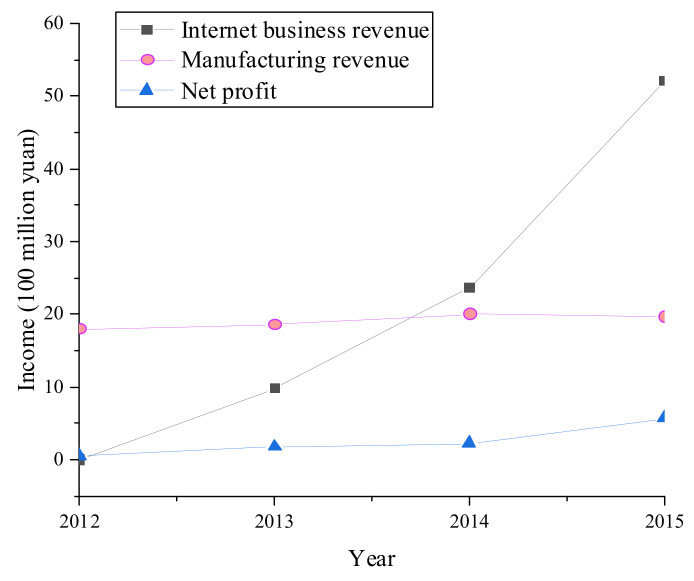
Changes in enterprise financial data during BYD’s EDT.

**Figure 9:**
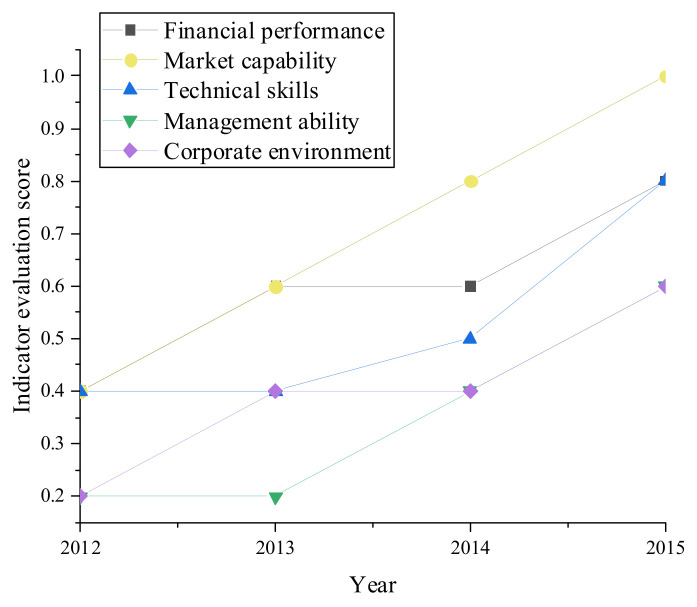
Changes in enterprise indexes during BYD’s EDT.

According to the financial changes of BYD during EDT from 2012 to 2015, with the growth over time, BYD’s Internet business revenue is far greater than the manufacturing revenue and net profit. Before EDT, BYD’s financial revenue mainly came from manufacturing and net profit. After EDT, BYD’s Internet business revenue accounts for 67.29% of the total revenue. Thus, EDT improves the revenue of manufacturing enterprises and reshuffles the revenue structure. Figure 8 manifests the enterprise financial data during EDT. Figure 9 portrays the changes in enterprise indexes during EDT.

According to Figure 9, all indexes have generally shown an upward trend during EDT, and the overall index increased by 42.11% from 2012 to 2015. From the change of the single index, the enterprise’s financial performance, market capability, and technical capability have been greatly improved after EDT. Therefore, EDT has absolute adaptability to BYD and greatly improves BYD’s index scores.

### 3.2. Analysis of Worker Health Status under the Proposed WHSM System

**Figure 10:**
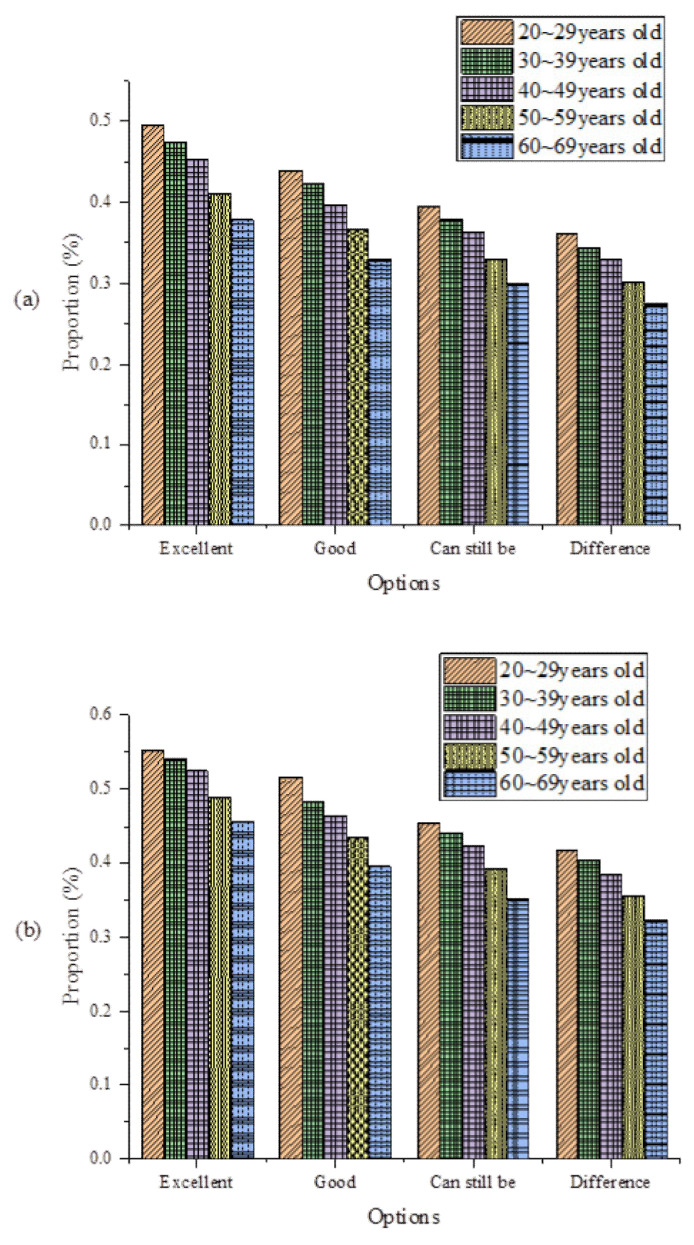
Analysis of cardiopulmonary capacity and cardiovascular health status of workers at different ages in BYD (**a**) before WHSM; (**b**) after WHSM.

During EDT, BYD has carried out intelligent and comprehensive management of workers’ health. Figure 10 plots the analysis results of the cardiopulmonary capability and cardiovascular health status of workers at different ages in BYD’s EDT.

Apparently, after WHSM, workers’ cardiopulmonary capability and cardiovascular capability have been improved. Therefore, the WHSM system can help improve workers’ heart and cardiovascular health and effectively avoid sudden diseases during work.

## 4. Conclusions

This work conducted a case study on BYD using the proposed EDT model and WHSM system by considering the adaptation and performance of the enterprise and the health status of workers. The results show that from 2012 to 2015, the Internet business revenue of BYD in the EDT process is much greater than the manufacturing revenue and net profit. After EDT, the Internet business revenue reaches 29.67% of the total revenues. Each enterprise index generally shows an upward trend, and the overall index has increased by 42.11%. Therefore, digital transformation has absolute adaptability to BYD manufacturing enterprises. The scores of various indicators of the whole enterprise have been greatly improved, especially the financial performance, marketability, and technical ability of the enterprises after transformation. In addition, after the enterprise’s digital transformation, employees’ cardiorespiratory and cardiovascular capabilities have been improved through employee health management. The findings provide important theoretical support for applying BDT and IoT in EDT and model references for the manufacturing industry’s EDT and WHSM. The proposed EDT model and WHSM system are based on the assumption that the attributes of manufacturing enterprises and other digital construction status quo share the same background. Thus, the proposed model and system cannot be applied to other industries. It is hoped that future research will improve the digital transformation mode of each enterprise.

## Data Availability

The further data can be reached by contacting the correspondence author.
